# LLMs and AI Life Models for Traditional Chinese Medicine-derived Geroprotector Formulation

**DOI:** 10.14336/AD.2024.1697

**Published:** 2025-04-09

**Authors:** Fedor Galkin, Feng Ren, Alex Zhavoronkov

**Affiliations:** ^1^Insilico Medicine AI Limited, Abu Dhabi, UAE.; ^2^Insilico Medicine Hong Kong Ltd., Hong Kong Science and Technology Park, Hong Kong SAR, China.; ^3^Insilico Medicine Shanghai Ltd., Shanghai, China.; ^4^Insilico Medicine, Cambridge, MA, USA.

**Keywords:** aging, traditional chinese medicine, geroprotectors, drug discovery, generative AI, multiagent system

## Abstract

Traditional Chinese Medicine (TCM) represents a vast repository of therapeutic knowledge, but its integration with modern drug discovery remains challenging due to fundamental differences in theoretical frameworks. We developed an AI agent-driven framework combining Precious3GPT (P3GPT), a multi-omics transformer model, with the BATMAN-TCM2 database of TCM compound-target interactions to bridge this gap. As a proof-of-concept, we used P3GPT-generated cross-species and cross-tissue signatures to screen TCM compounds, herbs, and formulas to identify novel natural geroprotectors. The cross-species analysis identified 13 conserved aging-associated genes, leading to the identification of 34 TCM compounds with significant target overlap and enabling identification of HUA SHAN WU ZI DAN and other TCM formulations as a promising historical formula. Our work demonstrates the feasibility of using AI to systematically bridge TCM and modern pharmacology, enabling rational design of multi-component formulations targeting age-related processes across multiple tissues and species. This approach provides a framework for modernizing traditional medicine while maintaining its holistic therapeutic principles. To help other teams integrate AI experimentation in their research process, we publicly release all materials and codebase used in this work, including the multi-agent system, cross-species and cross-tissue signatures of aging, as well as TCM databases formatted for AI interactions.

## INTRODUCTION

Traditional Chinese Medicine (TCM) encompasses a set of clinical practices rooted in a distinct historical tradition, such as acupuncture, breathing techniques, meditation, exercise regimens, moxibustion, massage, and herbal pharmacology, which are commonly combined to enable a systemic effect. All these practices are intended to normalize the flow of the vital energy (qi) through body meridians and restore the balance of elements governing organismal functions, such as wind, fire, dampness and others. Diseases in TCM are conceptualized as internal or external influences disturbing the balance of these elements in the total organism or specific meridians. The herbs used in TCM pharmacology are aligned with specific elements and their administration is expected to restore the balance disturbed by an affliction.

In contrast with modern Western pharmacology that operates with isolated compounds, applying herbal mixtures featuring dozens of active compounds is one of the core principles of TCM therapeutics. Any TCM drug is thus inherently multi-target which poses a challenge to anyone attempting to dissect the underlying biological processes. While some attempts have been made at formally describing the mechanisms of action encountered in TCM herbology, the existing compositions are mostly derived from historical sources [1-3].

TCM dispensatories offer tens of thousands of empirically derived formulas over the millennia of practice. Today, herbal mixtures mentioned in hundreds of medical texts have been extensively catalogued by research teams studying TCM in the context of modern clinical approaches [3, 4]. Efforts such as TCMSP, TCMIP, and BATMAN-TCM listing individual compounds, associated protein targets, and indications are particularly useful for advancing our understanding of TCM mechanisms [5, 6]. As some authors highlight, *in silico* methods of drug screening are an essential component of modern TCM studies since they enable cost-effective strategies of herbal medicine development [7].

With the rise of large language models and generative AI, *in silico* experimentation has reached a new level. The first biomedical AIs such as BioGPT were trained on published academic texts to answer domain-specific questions as those encountered in diagnostics [8]. More recently, a new type of biomedical AI started to emerge trained to operate with low-level biodata annotated with age that we call “life models” to separate them from natural text-based specialist models. Thanks to inventive data representation and training procedures, life models can interpret -omics experiments and learn biological processes that have not been covered in academic literature. Their ability to extract previously undescribed knowledge from raw experimental data makes life models more powerful epistemic engines than traditional LLMs, which can only recombine facts already stated in literature. [9].

Life models are a rapidly developing class of AI systems with such notable representatives as Geneformer trained on a corpus of 95MM single-cell RNA sequences, CpGPT and MethylGPT trained to DNA methylomes, and multi-modal Precious1GPT, Precious2GPT, and Precious3GPT (P3GPT) trained on multiple omics data types [10-16]. Among them, P3GPT is the only one capable of emulating the response of cells and tissues to various perturbators, including small molecules, clinical conditions, and aging. Serving as reliable approximations of living systems, life models open new avenues for swift and affordable anti-aging drug development [11]. Most importantly, P3GPT has been successfully used to identify novel geroprotectors, such as maslinic acid, XL-888, and others whose activity was validated *in vitro* [11]. In the present work, we demonstrate novel AI-based workflows of TCM formula composition and propose several herbal formulas for further validation of their anti-aging benefits. By combining P3GPT's ability to generate tissue-specific aging signatures with comprehensive TCM databases, we enable systematic screening of herbal compounds and formulas for geroprotective effects across multiple tissues and species. The workflows are publicly released on Github and may be adjusted to support TCM geroprotector discovery targeting multiple tissues, age groups, and experimental settings, thereby providing the research community with a computational framework for rational design of TCM formulas.

## MATERIALS AND METHODS

### Generative omics

We used a publicly available generative AI model Precious3GPT, deposited at HuggingFace (https://dx.doi.org/10.57967/hf/2699) [11]. P3GPT is a transformer-based AI model trained on a corpus of 1.2MM transcriptomic, epigenetic, and proteomic experiments, encompassing three species (human, mouse, macaque) and 300 tissues. Supplied with appropriate instructions, it emulates a corresponding omics experiment to provide differentially expressed features. Currently supported instructions include age2diff for cross-age group studies, dis2dif for studies of medical conditions, and cpd2diff for chemical screening experiments.

### Aging signature generation

We propose two ways of defining the aging process to support digital geroprotector screening experiments. Both workflows are available in P3GPT’s public Github repository precious3-gpt (https://github.com/insilicomedicine/precious3-gpt).

First, P3GPT was instructed to generate differentially expressed genes when for (i) human samples of 20-25- and 70-80-year-old individuals, or (ii) murine samples of 20-30- and 350-400-day-old individuals. These particular age groups were selected based on higher representation in P3GPT’s training set compared to more extreme age groups. Within each arm, 100 up-regulated genes were generated for each of the following tissues: adipose, skin, liver, muscle, lung, heart, and kidney.

Then, we selected the genes that may be responsible for the aging mechanisms preserved across species by intersecting gene lists from human and murine generations. The alternative approach of intersecting gene lists within human generations from different tissues was expected to generate aging hallmarks more relevant to human biology.

The enrichment analysis of the genes shared by multiple species was carried out via the Enrichr KG platform (https://maayanlab.cloud/enrichr-kg), and the raw output of these analytics are available in Supplementary File 2 [17].

### TCM database construction

We reformatted previously published BATMAN-TCM2 online database documenting protein targets of compounds encountered in TCM and deposited it on Hugging Face (https://dx.doi.org/10.57967/hf/3314) for easy public access as batman2 [6]. The database contains 118,176 cross-references entities, such as TCM formulas, herbs, and individual compounds.

To enable agent-based descriptions for the formulas designed using the developed software, we applied AI agents to harmonize web-sourced TCM materials and annotate them with SNOMED terms to enable unambiguous mapping between herbs, formulas, and medical conditions. The resulting database was deposited on Hugging Face (https://doi.org/10.57967/hf/3557) as the DragonTCM dataset, featuring detailed cross-referenced descriptions for 4,743 TCM entities [18].

We additionally connected identical entities between batman2 and DragonTCM to extend the utility of the combined dataset.

### TCM formula design

Our setup has allowed us to seek compounds based on their desirable targets, which were defined using P3GPT as aging-associated genes. To identify candidate compounds to be included in herbal formula targeting aging, we applied Fisher’s exact test (p-value < 0.01 after multiple comparison correction) of enrichment between compounds’ targets as specified in batman2, and P3GPT-generated signatures of aging. In these statistical tests, we used the total number of genes P3GPT could generate (N=25,332) as the background set.

We further filtered the compounds to select only those that are expected to affect multiple aging signatures. Our setup then allowed us to screen all batman2 herbs and formulas and select those that contain a larger subset of sought compounds. For custom formulas, we additionally placed constraints on animal-derived TCM components and the total number of herbs.

### Agent-derived formulas recommendations

The demonstrated framework for an agent-based TCM recommendation system was implemented using just-agents (https://github.com/longevity-genie/just-agents) Python library supporting any LLM with API access. The agents defined in the framework are supplied with tools enabling the retrieval of information on TCM entities to increase the accuracy and relevance of generated formulas and recommendations.

We based the agent system on the Rule of Four commonly used in TCM formula composition [2]. This empiric rule designates four roles the herbs in a formula need to play: Jun (Monarch) is responsible for the primary therapeutic action, Chen’s (Minister’s) role is to directly target secondary symptoms not covered by Jun and enhance its effects. The other two herbs in the Rule of Four act as compensatory elements: Zuo (Assistant) is selected to attenuate any adverse effects caused by Jun or Chen, while Shi (Envoy) restores the balance of the overall formula and guides its activity toward a particular meridian.

We adopt this TCM guideline by implementing an AI agent for each of these roles and adding an additional agent responsible for reviewing the toxicity and adverse effect risk. The input to the agents is provided as a patient-filled form stating the age, sex, medical conditions, and complaints. Jun and Chen agents select an herb from the top-20 herbs ranked based on their target’s intersection with P3GPT-defined gene lists. Zuo and Shi agents select the herbs based on their temperature, taste, meridian specificity, and potential toxic effects of Jun and Chen.

The agents have access to the tools that allow them to inspect detailed annotations on the pre-selected herbs in the attached databases (DragonTCM and BATMAN-TC2). Furthermore, Jun and Chen agents retrieve pathway enrichment scores for the targets affected by each considered herb via Enrichr [17].

As an additional measure to ensure formula safety, each agent-selected herb is reviewed in the context of the user-provided health information by the Contraindication agent, who can veto an herb, thus initiating a new round of selection by a Rule of Four agent.

Finally, the Designer agent is provided with user input and annotations for all passing herbs to present a formula with recommended dosages, course duration, and a breakdown of the affected molecular pathways and meridians. The code to replicate this agentic pipeline is available at the project’s Github repository (https://github.com/Insilico-org/TCMxPlore).

### Code availability

TCM datasets formatted for AI interactions are available at Hugging Face Datasets as batman2 (https://dx.doi.org/10.57967/hf/3314) and dragonTCM (https://doi.org/10.57967/hf/3557). The P3GPT model is available at Hugging Face as precious3-gpt-multi-modal (https://doi.org/10.57967/hf/2699). The code for the agentic TCM screening system and TCM database interactions is available at Github (https://github.com/Insilico-org/TCMxPlore). At the time of submission, a demo application based on the methods described in this article is available at a public Discord server (https://discord.gg/P4PWFNbYFg).

**Figure 1.**
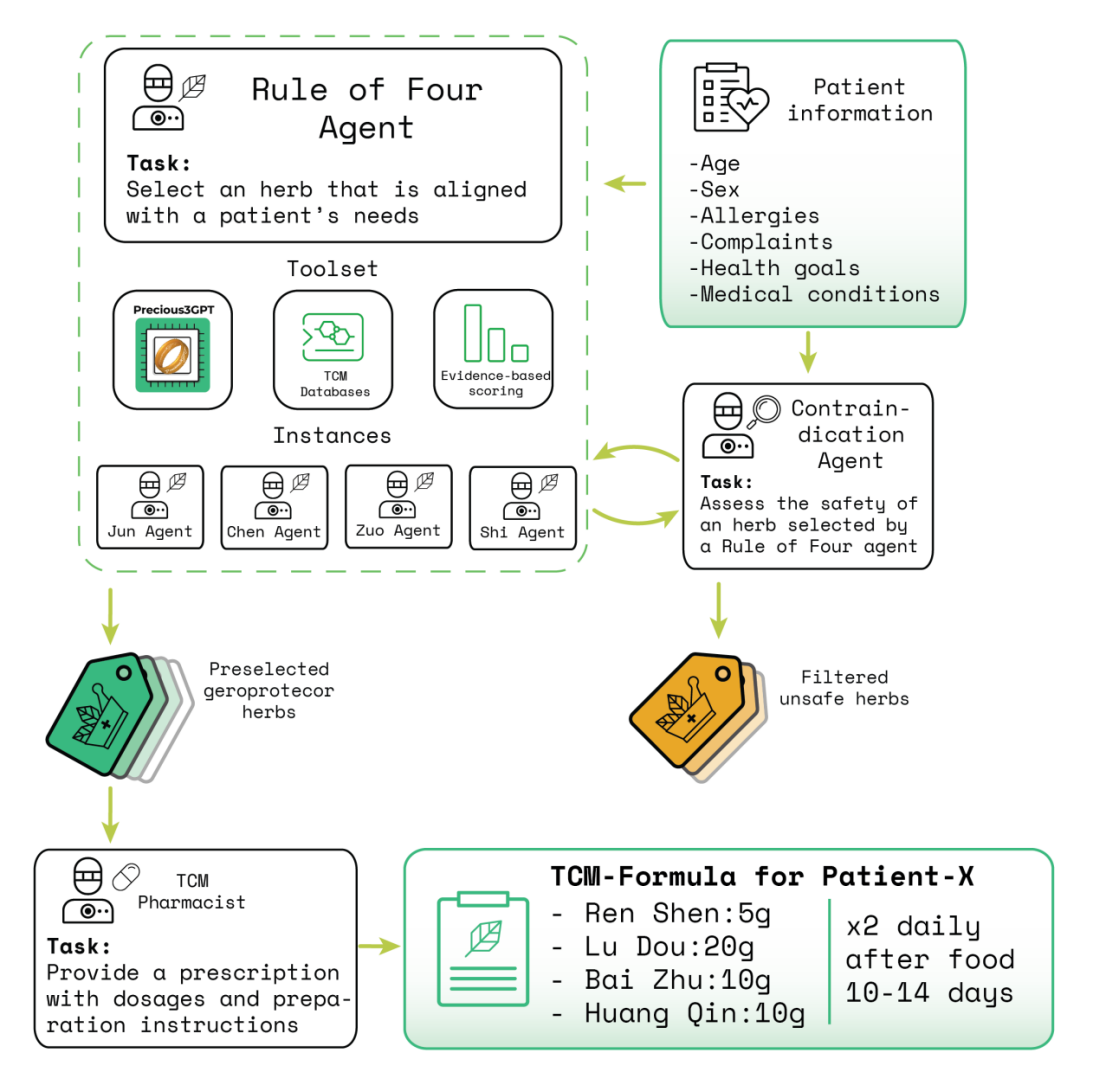
**Workflow for identifying cross-species TCM geroprotectors using Precious3GPT**. Precious3GPT was used to generate tissue-specific aging signatures across 7 tissues, comparing young versus old samples in both humans and mice. For each tissue, the model generated lists of 100 genes upregulated with age. The cross-species analysis focused on liver (33 genes genes shared between humans and mice), lung (27 genes), and muscle (22 genes) tissues, identifying 13 genes present in at least two tissue signatures. These genes were used to screen the BATMAN-TCM2 database for compounds with significant target overlap (Fisher’s test P<0.01). The analysis identified 90 compounds affecting liver aging signatures, 25 affecting lung signatures, and 27 affecting muscle signatures, with 34 compounds showing effects across multiple tissues. Formula screening identified HUA SHAN WU ZI DAN as containing the largest subset (20/34) of these compounds. Additionally, individual herb screening based on the 13 cross-tissue genes identified Astragalus, Cistanche, and Hawthorn as potentially beneficial herbs for targeting age-related changes across multiple tissues.

## RESULTS

### Cross-species signatures of aging

To initiate the quest for TCM herbs and formulas that carry anti-aging potential, we defined what constitutes aging using the P3GPT transformer model. Trained in a collection comprising 1.2MM omics experiments, this model provides differentially abundant omics features for a set of supported perturbators, one of them being the passage of time.

More specifically, we formatted P3GPT's prompts to emulate case-control RNA expression observations in young versus old humans and mice across multiple tissues (Fig. 1). The generated signatures containing 100 differentially expressed genes were analyzed to identify (i) single tissue cross-species signatures of aging, and (ii) multi-tissue human signatures of aging. The gene lists of the identified aging signatures are available in Supplementary File 1.

In one of the AI-driven geroprotector discovery strategies, we focused on three organs actively discussed in TCM literature: liver, lung, and muscle — and only considered the genes upregulated in the old [19-21]. Aiming to select formulas that may be studied in animal models, only the genes present in both human and murine aging signatures were considered. The resulting gene lists were inspected with pathway enrichment analysis tools to confirm the validity of P3GPT’s output (see Table 1, Supplementary Files 1, 2).

**Table 1:** Genes whose transcription levels increase in the aging process in both mice and humans, according to P3GPT.

Tissue	Gene list (ordered by importance)	N genes
**Liver**	APOA1; CYP2C19; CPS1; ANKRD1; SLC34A1; CYP3A7; CD1A; CMPK2; ACSM1; DHRS2; MIOX; ABCB11; TKTL1; UGT3A2; FABP1; KRT15; SERPINA3; SRPX; CRP; SCD; TIMP4; C3; AHSG; NNMT; MT-ND4L; OVCH1; FMO3; SLC46A1; CFI; DDIT4; LBP; PIGR; LEP	33
**Lung**	PLEKHG1; LRP1B; SLC17A7; FAM107A; SHISA2; ABCB1; MLPH; SLC5A9; ACSM1; SGMS2; PTPRG; SLC13A3; SCD; SERPINA3; SRPX; C3; SLC7A2; C4BPB; MT-CO1; B3GALT1; NNMT; OVCH1; TNR; MOBP; PZP; LRRC32; CFD	27
**Muscle**	MYH1; CYP2B6; MYH15; CYTL1; SLC22A7; KYNU; HOXA13; LYPD2; ACSM1; FBP2; MIOX; KRT15; COL1A1; SLC7A2; HBD; GABRP; MT-ATP8; APOD; MOBP; KDM5D; LBP; CFD	22

See all the available gene lists in Supplementary File1, see the results of enrichment analysis in Supplementary File 2.

The lung cross-species signature includes multiple carcinoma-associated genes (TNR, SCD) and, to a lesser extent, genes involved in complement deficiency (CFD, C3) and NAD+ metabolism (NNMT), all of which may be considered aging-associated processes [22-24].

In the liver signature, we detected genes involved in hepatic tissue identity (FABP1, APOA1, AHSG), some of which overlap with genes involved in hepatosteatosis (FABP1, AHSG, CRP). The signature also features inflammation mediators (CRP, LBP, AHSG), which might represent the inflammaging aspect of liver aging [25, 26].

The muscle cross-species aging signature features muscle-specific genes (APOD, LBP, COL1), inflammatory genes (CFD, LBP, KYNU), and genes involved in nutrient metabolism (APOD, FBP2, ACSM1, KYNU).

Notably, several genes appear in signatures across multiple tissues, possibly indicating evolutionarily conserved aging mechanisms linked with inflammation (C3, LBP, SRPX), metabolism (NNMT, ACSM1, MIOX, SCD), and extracellular matrix maintenance (SERPIN3). By focusing our TCM compound screening on these conserved aging-associated genes, we establish a systematic approach to identify herbal formulas that can be validated through parallel preclinical studies in mice and subsequent clinical trials in humans. This cross-species strategy increases the likelihood of discovering geroprotective formulas with translational potential.

### Cross-species TCM geroprotectors

To identify TCM formulas with cross-species anti-aging potential, we implemented a systematic bottom-up approach using two complementary strategies: analysis of existing formulas and design of novel herb combinations. Both strategies were based on cross-referencing aging-associated gene signatures with the BATMAN-TCM database of compound-target interactions.

First, we employed Fisher's exact test with multiple comparison correction (P-value < 0.01) to identify natural compounds whose molecular targets significantly overlapped with the cross-species aging signatures. This analysis revealed 90 compounds affecting the liver signature, 25 affecting the lung signature, and 27 affecting the muscle signature (Supplementary File 3). Among these, 34 compounds showed significant effects across at least two tissues, suggesting their potential role in systemic aging processes.

We then screened the BATMAN-TCM2 database for formulas containing these compounds. While no single formula contained all 34, HUA SHAN WU ZI DAN, HSWZD) emerged as the most promising composition, incorporating 20 of them. HSWZD, first documented in the 1594 manuscript "Lu Mansion's Secret Recipes", comprises 27 herbs including ginseng, aconite, rehmannia, atractylodes, and ephedra, along with medicinal fungi such as Poria cocos and Ligusticum striatum [27]. Among the 20 compounds included in HWZD, are those with known health benefits when used as dietary supplements: butyric acid [28, 29], arachidic acid [30], deoxycholic acid [31], and vitamins (ascorbate, tocopherol). However, our analysis also identified potentially harmful compounds in this formula, such as hypoxanthine, which has been associated with muscle fatigue and dyslipidemia [32, 33].

**Figure 2.**
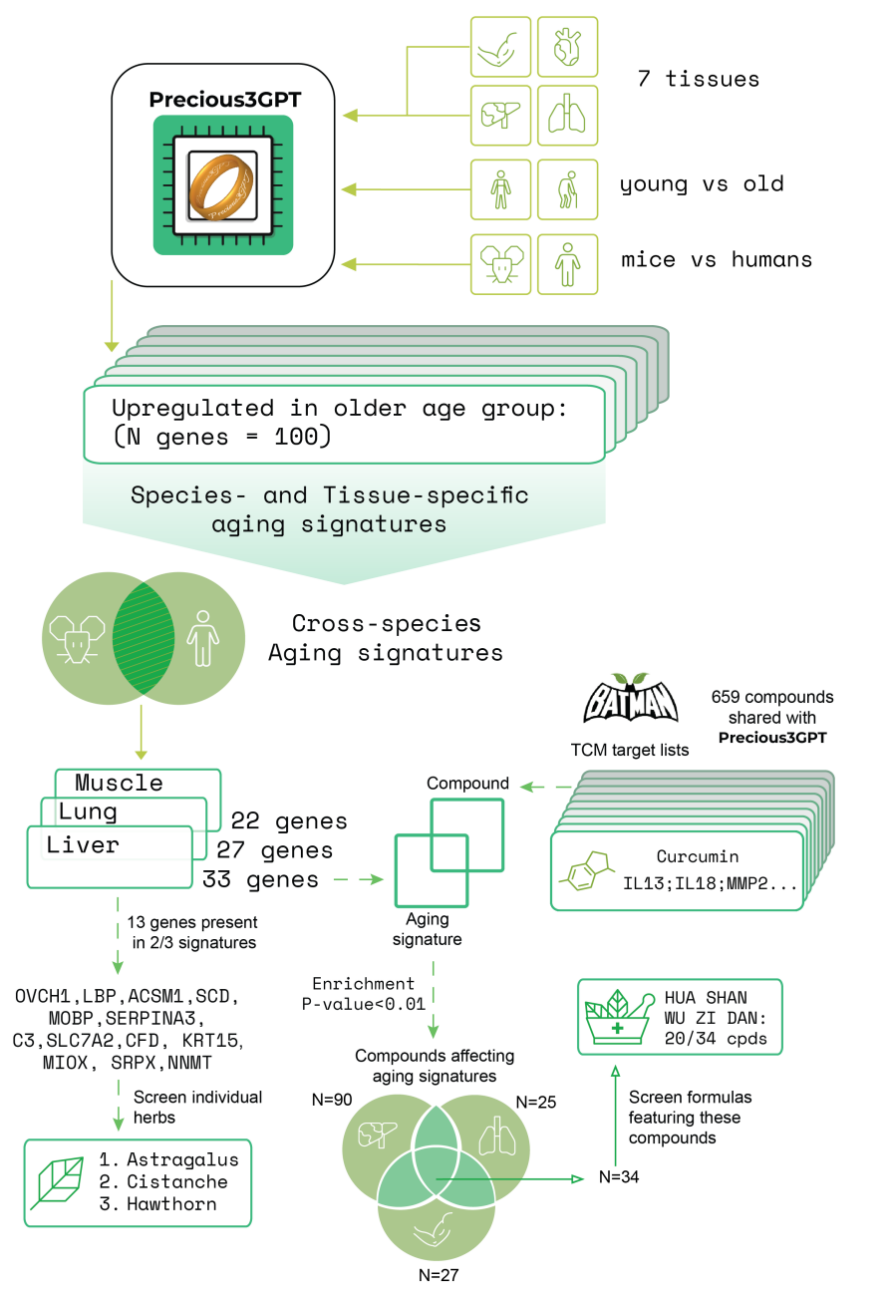
**Multi-tissue screening approach for TCM compounds and formula design**. (**A**) Precious3GPT was used to generate aging signatures for seven human tissues: adipose, heart, kidney, liver, lung, muscle, and skin. These signatures were screened against the molecular target of compounds in the BATMAN-TCM2 database to identify potential geroprotectors. (**B**) Distribution of 294 compounds based on the number of tissue aging signatures they significantly affect (Fisher’s P<0.001). 54 compounds showed effects across all seven tissues and were used to design two formulas: ISM-Formula#1, containing 30 herbs and capturing 53 of the 54 compounds, and ISM-Formula#2, a simplified version with just 4 herbs (Ginseng, Tea leaves, Ephedra, and Sainfoin) capturing 21 of the target compounds despite much simpler composition.

Instead of screening existing formulas, one may also screen individual herbs to compile a new formula. We identified 13 genes present in at least two cross-species aging signatures: OVCH1, LBP, ACSM1, SCD, MOBP, SERPINA3, C3, SLC7A2, CFD, KRT15, MIOX, SRPX, and NNMT. By screening BATMAN-TCM2's collection of 24,666 herb-compound-target annotations, we identified three herbs containing the highest number of compounds targeting these genes: SHA YUAN ZI (Astragalus seed), ROU CONG RONG (Cistanche leaves), and SHAN ZHA YE (Hawthorn berry). While these herbs have not been previously combined in traditional formulas, they are all considered safe for consumption and may potentially work synergistically to promote healthy aging across multiple tissues.

Our dual analytical approach identified both a historical formula (HSWZD) and a novel three-herb combination targeting cross-species aging signatures, demonstrating the potential of computational methods to bridge traditional and modern pharmacology. While HSWZD's composition raises some safety concerns, the alternative combination of Astragalus seed, Cistanche leaves, and Hawthorn berry offers a potentially safer starting point for developing systemic geroprotective interventions.

### Human multi-tissue TCM geroprotectors

Building upon our cross-species analysis, we explored an alternative P3GPT-supported strategy focused on identifying compounds that affect multiple tissues within a single species (Fig. 2A). We expanded our tissue panel to include skin, heart, kidney, and adipose tissues in addition to the previously analyzed tissues, then systematically evaluated natural compounds from P3GPT's training set based on their targets’ intersections with tissue-specific aging signatures.

This analysis identified 294 compounds affecting at least one tissue-specific aging signature, with 54 compounds showing significant overlaps with all seven tissues (Figure 2B, Supplementary File 4). These multi-tissue compounds include established geroprotectors (resveratrol, nicotinamide), hormones (progesterone, estradiol, prednisolone, hydrocortisone, melatonin), vitamins (retinol, tocopherol, ascorbate, pyridoxin, cobalamin), and other bioactive molecules like caffeine, theanine, and aspirin.

When screening existing TCM formulas, we found that TOU GU ZHEN FENG WAN (TCMFx5163 in the TCM-ID database [34]), a 69-herb formula traditionally used for inflammatory joint conditions, contained the highest number of these compounds (25 out of 54). Recent clinical evidence shows this formula outperforms glucosamine sulfate in treating osteoarthritis [35], suggesting it may have broader anti-inflammatory effects that counter the inflammaging processes in multiple tissues.

**Figure 3.**
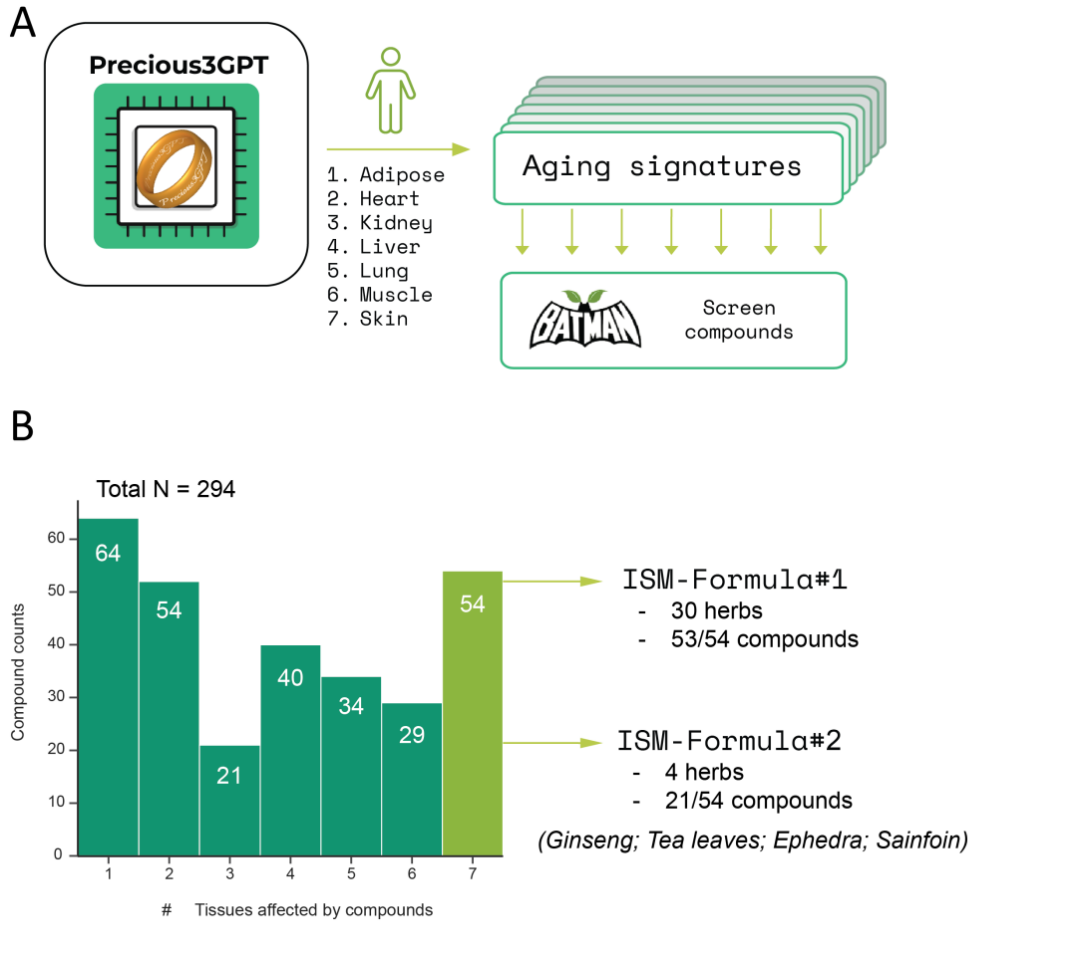
**Multi-agent system for personalized TCM formula development**. The workflow presented in this article is a collaborative AI system for designing and validating personalized TCM formulas. Four herbs are selected sequentially by different agents based on a combination of molecular target scores, TCM guidelines, and available patient information. Each agent is equipped with tools to access detailed annotations on top-scoring herbs to ensure informed decision-making. All herbs to be included in the final formula are reviewed by an agent for potential contraindications and removed from consideration if they can lead to adverse effects in the patient. Finally, the TCM Pharmacist agent integrates all the retrieved information to generate an annotated prescription, specifying exact dosages, preparation, and treatment instructions. You may find these agents in our GitHub repository (https://github.com/Insilico-org/TCMxPlore).

To capture more of the identified compounds, we developed two novel formulas using different design principles. First, we employed a greedy algorithm to create ISM-Formula#1, comprising 30 herbs that collectively contain 53 of the target compounds (detailed in Supplementary File 5). Then, drawing from the traditional Four Roles principle [2, 36], which emphasizes balanced formulations with at least four key components, we created ISM-Formula#2. This simplified version contains just four herbs: ginseng (REN SHEN), tea leaves (CHA YE), ephedra (MA HUANG), and sainfoin (LU DOU), yet still captures 21 of the target compounds.

This human-only multi-tissue strategy demonstrates a novel computational approach for geroprotector discovery by leveraging P3GPT. The identification of 54 compounds affecting all tissues not only validated known geroprotectors but also enabled the rational design of two promising formulas.

### Agent-based formula composition

While our computational approach successfully identified promising herbal combinations, it has notable limitations: it cannot personalize formulas based on individual patient needs, specify precise dosages, or interpret the formulas' mechanisms of action through traditional TCM principles.

To address these limitations, we have developed a multi-agent system integrated with online TCM resources (Fig. 3). The system comprises six specialized agents that work collaboratively to pick and review the herbs that fit a person’s profile the best. The agentic system sequentially adds Jun, Chen, Zuo, and Shi herbs (see Methods), checking their safety in the context of user-provided input. Aided with detailed annotations from the attached databases, our MAS can provide highly personalized and safe formulas with.

The MAS may be inspected in more detail at our GitHub repository (https://github.com/Insilico-org/TCMxPlore) and public Discord channel (https://discord.gg/P4PWFNbYFg).

## DISCUSSION

The practice of isolating single therapeutic compounds, a cornerstone of modern pharmacology, emerged relatively recently in medical history - primarily in the XIX^th^ century, when advances in organic chemistry and physiology first enabled systematic chemical isolation and animal experimentation [37]. This stands in stark contrast to TCM, which has evolved over three millennia and emphasizes the therapeutic use of complex herbal mixtures within a holistic treatment framework [38].

While TCM and other herbal medicine traditions are often dismissed as pre-scientific or obsolete, such criticism may reflect a modern bias that overlooks the value of accumulated clinical experience spanning thousands of years [39]. The historical record demonstrates that many breakthrough medications originated from traditional herbal remedies. Digoxin, derived from *Digitalis lanata* [40], morphine from *Papaver somniferum* [41], and aspirin from *Salix alba* [42] represent just a few examples of modern pharmaceutical developments built upon traditional herbology. Perhaps the most compelling case is Tu Youyou's 1972 discovery of artemisinin, which earned her the 2015 Nobel Prize [43]. By consulting ancient TCM texts, she identified not only promising anti-fever formulas but also crucial extraction methods that led to this potent antimalarial compound. More recently, cycloastragenol extracted from a medicinal herb, *Astragalus*, has been identified as a telomerase activator with therapeutic potential in lung fibrosis and anti-aging treatments [44-46]. As illustrated by these examples, the synthesis of herbal medicine and modern pharmacology can yield the most exciting results.

Despite this impressive record, herbology and TCM remain underutilized resources in modern drug development. Our work addresses this missed opportunity by developing a novel approach that combines generative AI with online TCM databases, creating a framework for identifying promising herbal formulations. While we focused on aging-related applications, this proof-of-concept methodology could be adapted to various therapeutic areas.

The potential of natural compounds in aging research is already well-established. Compounds like resveratrol, curcumin, berberine, and quercetin have been extensively studied for their geroprotective properties [47]. However, these are but a fraction of the herbal compounds that could affect the aging process. Given the vast number of herbs used in TCM and compounds featured in them, there may be numerous undiscovered natural compounds to be developed into anti-aging interventions [48, 49]. Our AI-driven approach offers a systematic method to explore this chemical space more efficiently than traditional screening methods.

We contribute to this exploration by presenting the community with tools to accelerate research projects in herbal medicine. The cross-species hallmarks of aging identified through our approach align well with established aging mechanisms, including chronic inflammation, extracellular matrix deterioration, and metabolic dysregulation [50]. This alignment with known biology supports the validity of our methodology while also revealing novel insights.

Particularly noteworthy among our findings is the identification of NNMT as a cross-species hallmark gene. This discovery is especially relevant given NNMT's role in NAD+ metabolism, whose precursors such as NMN and NR are being actively investigated as potential anti-aging interventions [51]. The importance of NNMT is further backed by the findings of its overexpression in malignancies and its association with reduced intracellular concentration of NAD+ [52-54].

Another significant finding from our cross-species analysis is ACSM1, which appears in multiple aging signatures. Recent research has characterized ACSM1 as both a potential oncogene and a suppressor of ferroptosis, with its overexpression promoting oxidative stress resistance [55, 56]. These findings suggest a complex role for ACSM1 in cellular stress response and aging processes.

A wide prevalence of inflammation-associated genes among all three aging lists highlights the potential benefits antioxidant and anti-inflammatory phytochemicals may have on a systemic level. Plant glycosides, such as those contained in ginseng, have extensive evidence of ROS scavenging activity and interactions with the NF -kB pathway [57]. These properties of ginsenosides have been linked to their beneficial effects in the context of oncological, cardiovascular, neurodegenerative, and metabolic diseases [58, 59].

We demonstrate several ways to compile and screen TCM formulas using P3GPT-generated aging signatures. The method that led us to the identification of HSWZD as a potential herbal geroprotector relied on selecting the compounds whose molecular targets intersect with P3GPT gene lists. In this approach, some selected compounds, such as hypoxanthine, have been reported to have negative effects on muscles [32]. We believe that such false positives may be improved by considering the mechanism of action of natural compounds, which is not yet available in BATMAN-TCM2.

An alternative approach relies on picking individual herbs to maximize the number of compounds targeting genes in the intersection of multiple cross-species signatures. The herbs we selected this way (astragalus, cistanche, hawthorn) have been extensively described in publications on herbal medicine. As such, the Astragalus seed extract has been reported to have beneficial effects in cardiovascular diseases, diabetes, and oncology [60]. Meanwhile, *Cistanche* extracts have been studied for their ability to inhibit apoptosis in neurons, hepatocytes, and fibroblasts in murine disease models [61]. The fruit of *Crataegi sp*. (hawthorn) have been shown to improve dyslipidemias, cardiac output, and exhibit antioxidant activity [62].

One major component of the HSWZD formula is ephedra, which is among the most potent antioxidant herbs [63]. Identified by targeting P3GPT-simulated aging processes in muscle, lung, and liver, ephedra is extensively studied in the context of these tissues. It has been shown to exude a protective effect in models of chemically-induced liver damage [64]and COPD[65], as well increase muscle performance (albeit with conflicting evidence) [66, 67] and promote weight loss by increasing the basic metabolic rate [68, 69]. This shows the relevance of the herbal ingredients selected via the proposed workflow and proves the concept of designing herbal formulas with generative AI and low-level compound annotations.

Yet, ephedra is also a heavily regulated herb. Its key active compound, ephedrine, was banned by FDA in 2004 due to its adverse effects on the heart and nervous system manifested as anxiety, palpitations, hypertension, and more serious health events [70, 71]. Some TCM recipes contain even more toxic compounds. For reference, the daily dose of ephedrine in the referenced studies conducted in the 1990s is within 50-60mg, while aconitine from aconite in HSWZD can cause a lethal outcome even at such low concentrations (LD50<0.3 mg/kg) [72]. In more extreme cases, TCM formulas contain ingredients that have no therapeutic window at all and are harmful at any concentration. Many TCM formulas feature arsenic and lead, giving rise to the infamous phenomenon of “Chinese alchemical elixir poisoning” which claimed the lives of a surprisingly large number of Chinese emperors and nobles from the III^rd^ century BC to the XVIII^th^ century [73].

Safety and dosage considerations necessitate the review of the generated formulas, a task that we propose to carry out using AI agents. For patient-oriented applications, the assessment of an herb’s safety in the context of a patient’s medical history should be done at each decision point. Noteworthy, while testing the formula composition pipeline (Fig. 3) with a dedicated Contraindication Agent, herbs containing ephedrine or aconitine were immediately removed from consideration. This emphasizes that the compounds identified with AI-based scoring need to be carefully validated *in vitro* and *in vivo* to identify safe therapeutic windows.

MAS are a relatively old concept that has recently risen to prominence with the AI boom as a means to make LLMs more autonomous and applicable to complex, multi-stage problems [74, 75]. Unitary AI agents are LLMs provided with scripted tools that increase their performance in specialized tasks and reduce token expenditure. MAS are structures of such agents which enable sharing information, task partition, delegation, sequential execution and other behaviors emulating human decision-making and problem-solving processes. Best practices to create such structures are still actively discussed and the approaches range from “quantity over quality” to elaborate architectures governing agent planning and cooperation [76, 77].

Most importantly, AI agents are fully capable of leveraging the functionality provided by life models such as P3GPT to model biological processes. While P3GPT can generate synthetic omics data and simulate experimental conditions across multiple tissues and species, MAS can orchestrate complex research workflows by automating data collection, analysis, and validation steps. For instance, one agent can utilize P3GPT to generate aging signatures across different tissues, while another agent simultaneously searches databases like BATMAN-TCM2 for matching compounds, and a third agent evaluates safety profiles and potential contraindications. This orchestrated approach can significantly accelerate the drug discovery pipeline by enabling parallel processing of multiple hypotheses and automated validation of results. As demonstrated in the P3GPT validation studies, where the model achieved a 36% hit rate in identifying novel geroprotectors, combining this predictive power with autonomous agents could help researchers more efficiently navigate the vast space of possible therapeutic combinations [11]. Moreover, MAS can enhance P3GPT's utility by continuously updating its knowledge base with new experimental data and research findings ensuring continuous evolution of the model.

For the purposes of demonstration, we constructed a MAS that replicates the traditional process of formula composition commonly known as the Rule of Four, or Jun-Chen-Zuo-Shi. In this process, each herb is tasked with a particular aspect of TCM therapeutics, such as treating the primary syndrome or mitigating possible toxicity. In our MAS, four Jun-Chen-Zuo-Shi agents sequentially select an herb to add to the formula based on their molecular components, pathway enrichment scores, or compatibility with the user and each other. Additionally, each herb is reviewed by a separate agent tasked with eliminating any harmful components. Finally, all the herbs are reviewed in aggregate by another agent to provide dosages, instructions, and explain the resulting formula’s action form both traditional and molecular points of view. This MAS structure ensures informed and personalized formulas design and communicates its process in a user-friendly format.

In addition to the traditional and pathway enrichment approaches, our agents rely on P3GPT to define signature gene lists, and thus, extend the range of geroprotective TCM formulas to be detected. This has allowed us to empower the agent with the ability to select compounds and herbs affecting the aging mechanisms shared by different species or different human tissues.

In particular, we explored human cross-tissue signatures to compose two custom formulas that are expected to have beneficial effects on the aging process in seven tissues. While the designed formulas include previously mentioned herbs, such as ginseng and ephedra, they also contain tea leaves and sainfoin. Green tea extract has a long history of supplementation experiments which have shown it to improve cellular antioxidant capacity, blood lipid profiles, and body composition [78-80]. Sainfoin is mostly described as a foraging legume that reduces parasitism and prevents bloating [81, 82]. However, recent studies position it as a nutrient-rich crop for human consumption and outline more benefits on animal growth and meat growth attributed to its high anthocyanin content [83, 84].

The methodology described in this paper can serve as a template for further studies involving AI for biomedical research. It should be noted that P3GPT is not the only possible source of aging signatures. Other possible sources include various aging clocks that have recently gained widespread adoption as tools of biogerontological research [85]. Aging clocks are statistical models trained on a variety of biodata types, such as proteomics and epigenetics, to assess the intensity of the molecular aging processes in tissues and organisms [86, 87]. These models may be applied to identify the genes driving the aging process and thus provide direction for geroprotector design, a strategy already used by some pharmaceutical companies [88, 89]. Notably, these various sources of gene signatures are not mutually exclusive and may be combined in a single MAS to provide a multifaceted view of the aging process.

MASs offer the flexibility other methods of data analysis are not equipped with. However, we suggest that agentic data exploration is the most powerful when relying on white-box methods grounded in established knowledge. While RAG implementations, such as Chroma, are commonly used to make LLM output more relevant, they lack transparency. Another option to promote informed decision-making by a biomedical MAS is fine-tuning the underlying LLM on corpora of relevant data. This approach has most recently been embodied in TxGemma, a Gemma 2 variant that fine-tuned with the Therapeutics Data Commons dataset [90, 91]. While yielding impressive benchmark performance, TxGemma’s responses are not fully traceable to the specific bits of information that have led to its response. We propose an alternative in placing agents in an environment with uniformly formatted entity descriptions to ensure the traceability of their decisions.

This paper shows the feasibility of an AI-based TCM formula composition and geroprotector discovery. While it opens new directions for AI-driven drug discovery, no decisions should be made in absence of laboratory experiments validating the safety and efficacy of the selected compositions. Due to ethical reasons, any digital model of life can serve only as a preliminary step in the drug discovery process and the final decision to move forward needs a more robust evidence base.

The presented framework may further be expanded to improve various aspects of a downstream application. For example, adding support for toxicity databases will enable a more accurate assessment of potential adverse effects. Due to the modular design of the MAS, extra functionality can be added with little refactoring required as tools that form queries to a database or interact with predictive models. Another set of improvements to the workflow may be focused on increasing the involvement of a user in formula generation. Assuming the user is a professional TCM practitioner, they may wish to include additional herbs and formulas at the database level, or override automatic herb selection at certain points, as well as grant MAS access to their own records to align its decisions with their own experience.

The combination of agentic LLMs with curated database access yields a powerful setup that offers both flexibility and precision. We hope that the methodology presented in this article will inspire other teams of researchers to collaborate with MASs to explore the domains of drug discovery and TCM.

## Conclusion

In the present work, we demonstrate a novel conceptualization of TCM. We have adopted a generative AI model P3GPT to enable a bottom-up approach of designing medicinal formulas. Our approach leverages information on natural compounds’ protein targets to identify combinations of herbs that are expected to have a strong effect on generated signatures of aging. The described approach can be easily generalized to enable high-throughput digital screenings for novel herbal interventions for any medical condition.

The TCM formula discovery pipeline is further expanded with AI agents to add explainability and interpret the herbal compositions in terms used by TCM practitioners. We have provided public access to all materials and code used in this project to aid other researchers aiming to bridge the gap between modern Western pharmacology and TCM.

## Supplementary Materials

All generations, formulas, enrichment results, and compound lists used in this paper are available as an Open Science Framework repository (https://dx.doi.org/10.17605/OSF.IO/ZND3U).
